# Plant growth regulator applications and mechanisms for boosting rice productivity

**DOI:** 10.7717/peerj.21457

**Published:** 2026-07-01

**Authors:** Yushawu Zakaria Mohammed, Obed Kweku Sackey, Aaqil Khan, Abdul Kadir Issah, Fatahiya Iliasu, Naijie Feng, Dianfeng Zheng

**Affiliations:** 1 College of Coastal Agricultural Sciences, Guangdong Ocean University, Zhanjiang, China; 2 South China Center of National Saline-Alkali Tolerant Rice Technology Innovation Center, Zhanjiang, China; 3 Fishery College, Guangdong Ocean University, Zhanjiang, China

**Keywords:** Abiotic stress tolerance, Agronomic efficiency, Hormonal crosstalk, Nano-formulations, Precision agriculture, Yield enhancement

## Abstract

Plant growth regulators (PGRs) have emerged as an important tool for improving crop productivity and adaptation to intensifying environmental challenges, especially in rice. This review summarizes 209 publications (2000–2025) on the current developments in exogenous PGR applications across major growth phases from germination to grain filling. It reveals the principal findings on hormonal crosstalk, including abscisic acid-gibberellin (ABA-GA) antagonism, stress balancing, and genotype-specific multi-hormonal strategies. It outlines 10–40% yield improvements in conjunction with precision agronomy. We elucidate how PGRs regulate growth, development, stress tolerance, and yield through molecular and physiological pathways. Nano-formulations and genome-editing methods offer revolutionary potential, combining hormonal modification with genetic improvement and digital technologies for sustainable intensification. While emphasizing the important role PGRs play, this review also highlights context-specific effects and application risks, and emphasizes variety-specific, evidence-based protocols. Ultimately, this work charts a course for future research and precision application, and it marks a shift in paradigm to create a stronger, more sustainable rice production system.

## Introduction

Rice (*Oryza sativa* L.) is a vital component of the world’s food supply, playing a crucial role in the global food system and accounting for more than 21% of all dietary energy, which contributes to achieving global food security (Li et al., 2024; Yuan et al., 2021). Nevertheless, the anticipated future needs in food production, projected to grow by 40 percent in 2050, will continue to pose problems for existing production mechanisms (Zhang et al., 2024b). Rice is challenged by multifaceted constraints, such as climate variability, nutrient deprivation, and abiotic stress, which depress its yield and resilience (Castilla et al., 2021; Radha et al., 2023). These interconnected stressors share the need for integrative management approaches that would lead to an increase in productivity and adaptability.

The natural and synthetic analogs of phytohormones in the form of plant growth regulators (PGRs) are increasingly becoming accurate tools to suppress or induce plant physiology in different environmental conditions. These PGRs alter growth, gene expression, and stress responses through engagement with endogenous signaling networks, thereby providing a non-genetic means to enhance crop performance (Agudelo-Morales et al., 2021; Farman, Mushtaq & Azeem, 2019). Although PGRs have been widely used in agriculture in rice, there is little mechanistic knowledge on the effects of this action in rice because most studies have focused on other grain crops, including wheat and maize (Sun et al., 2025; Tang et al., 2024).

This review synthesizes recent developments in the application of PGRs to rice improvement, with special reference to genotype-specific responses, interactions between hormones, and their use under abiotic stress conditions. It emphasizes the transition of studying PGRs as individual components to considering them as parts of multi-faceted regulation networks and discusses their possible combination with new technologies, including nano-delivery, CRISPR editing, and digital agriculture for more precise and environmentally friendly crop management. An attempt is made in the review to set PGRs in the context of this systems-based approach with a view to developing roadmaps for improving rice productivity and its robustness, stating responsive actions for world agriculture.

### PGRs in rice enhancement

Various PGRs such as abscisic acid (ABA), auxins, gibberellins (GAs), cytokinins, ethylene, brassinosteroids (BRs), salicylic acid (SA), jasmonates, paclobutrazol, and uniconazole control rice development and adaptation to stress. These compounds, which alter metabolic activity and biochemical pathways, control the key developmental stages such as seed germination, tillering, panicle initiation, flowering, and grain filling (Elsisi et al., 2024; Rademacher, 2015; Yan et al., 2022). For instance, foliar application of kinetin increases yield components by increasing the metabolic efficiency (Zhang et al., 2021).

PGRs also enhance abiotic stress tolerance *via* interconnected signaling cascades that regulate antioxidant defenses, water homeostasis, and gene expression (Thilakarathne, Liu & Zou, 2025; Wani, 2023; Zahid et al., 2023). For instance, exogenous GA_3_ induces root and shoot elongation and decreases reactive oxygen species (ROS), and further increases potassium uptake and enzyme activity under stress conditions (Pal et al., 2023; Shahzad et al., 2021; Swain et al., 2023). Furthermore, ABA causes stomatal closure in drought conditions, and cytokinins and SA are responsible for increasing overall resilience as observed in rice during salinity and heat stresses (Li et al., 2017; Pantoja-Benavides, Garces-Varon & Restrepo-Díaz, 2022; Saradadevi, Palta & Siddique, 2017).

The efficacy of PGRs is highly dependent on the rice genotype, which dictates the type, optimal dose, and timing of application. This genotype-specificity is a fundamental reason why universal, one-size-fits-all PGR formulations are ineffective (Hussain et al., 2020). The responses of Indica and Japonica subspecies to GA_3_ and auxin differs. GA_3_ tends to cause etiolation and root lodging in Indica seedlings, and indole-3-butyric acid (IBA) induces active rooting in both species (Table 1) (Wahyuni et al., 2016). In contrast, cytokinins effectively enhance the yield of mainly high-yielding cultivars such as Basmati-385, but not of KS-282 (Jalal-ud-Din et al., 2015). Given the underlying genotype and environment interaction, it highlights the requirement for specific applications to optimize growth, minimize stress, and support sustainable productivity (Pal et al., 2023; Zahid et al., 2023).

**Table 1:** Comparative responses of major rice subspecies (Indica and Japonica) to common plant growth regulators. Comparative analysis of how major rice subspecies (Indica and Japonica) respond to key plant growth regulators (PGRs), including gibberellins, auxins, cytokinins, and ABA.

PGR class	Subspecies	Typical response & application consideration	Agronomic implication	Notable varietal examples	References
Gibberellins	Indica	Highly responsive; it is useful for overcoming seed dormancy and enhancing stem growth. Produce excessive elongation and lodging in tall, traditional varieties. Dose control is critical.	Enhances seedling vigor and panicle exertion.	All varieties, like IR64, show strong elongation.	Wang et al. (2021)
Gibberellins	Japonica	Generally less responsive than Indica; often already semi-dwarf due to altered GA pathways such as the sd1 mutation. This is mainly applied in seed priming or to enhance panicle excretion of some hybrids.	Reduces the risk of lodging while achieving desired panicle growth.	Semi-dwarf varieties like Nipponbare; application in hybrids like Akitakomachi.	Wang et al. (2013)
Auxins	Indica	Promotes vigorous rooting and robust seedling development. Its use is preferable to that of GA_3_ in the early stage of growth.	Improves establishment and transplant success.	Basmati-385, IR64	
Auxins	Japonica	Enhanced root biomass and lateral root development. Helps in improved absorption of nutrients under low nutrient soils.	Increases stress tolerance during early growth.	Nipponbare, Koshihikari	Cui et al. (2025), Mickelbart, Hasegawa & Bailey-Serres (2015)
Cytokinins	Indica	Strong yield response in high-yielding varieties; promotes tillering and grain filling.	Significant yield increases (10–20%) in responsive genotypes.	Basmati-385	Jalal-ud-Din et al. (2015), Jameson & Song (2016)
Cytokinins	Japonica	Variable tillering response; more effective under stress conditions than for yield alone.	Stress protection rather than primary yield enhancement.	KS-282 (weaker response)	Jalal-ud-Din et al. (2015)
ABA	Indica	Strong stomatal closure under drought; higher endogenous requirement than Japonica.	Critical for drought avoidance in rainfed systems.	Drought-prone Indica landraces	Li et al. (2017)
ABA	Japonica	Moderate stomatal response; genotype-dependent sensitivity.	Supports water use efficiency in temperate conditions.	Temperate Japonica varieties	Li et al. (2017)

## Methodology

**Figure 1:**
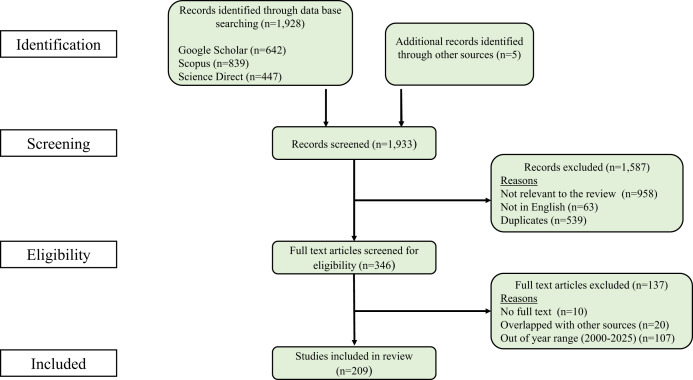
PRISMA flow diagram of literature identification, screening, eligibility, and inclusion. The four-stage process for identifying and selecting studies for the present review. The initial search across Scopus, Google Scholar, and ScienceDirect yielded 1,928 records, supplemented by five additional records from research webpages. After removing duplicates and screening titles/abstracts, 1,587 records were excluded due to irrelevance, language, or duplication. Of the 346 full-text articles assessed for eligibility, 137 were excluded for lacking full text, overlapping with other studies, or falling outside the specified publication year range (2000–2025). Ultimately, 209 studies met the inclusion criteria and were retained for qualitative synthesis.

The research relies on extensive empirical evidence extracted from several literature sources, using a systematic literature search and selection process to identify relevant studies on plant growth regulator applications in rice. The search and selection methods followed the PRISMA (Preferred Reporting Items for Systematic Reviews and Meta-Analyses) framework and are shown in Fig. 1.

### Search strategy and selection criteria

Key scientific databases (Scopus, Google Scholar, and ScienceDirect) were used to conduct the literature search. The search was purposefully restricted to articles within the time range of 2000–2025 to identify the latest developments in the field of molecular mechanisms, agronomic applications, and novel technologies such as nano-formulations and CRISPR-Cas9 that are most aligned with the modern agricultural challenges. Although we recognize the extensive history of PGR research, this time frame was chosen to emphasize studies that underscore current scientific knowledge and technical capabilities. The search terms used were combinations of keywords, including plant growth regulator, rice, *Oryza sativa*, abiotic stress, and sustainable agriculture.

### Study selection and data extraction

A total of 1,928 database records were obtained, from which duplicates were eliminated. The remaining records were screened according to their titles and abstracts to determine their relevance to the review’s objectives. The full texts of potentially eligible articles were then checked against pre-defined inclusion criteria (1) studies that applied exogenous PGRs to rice, (2) studies that studied physiological, molecular, or agronomic reactions, and (3) articles written in English. After this process, a total of 209 studies were included for qualitative synthesis in this review. Mendeley reference manager (Elsevier) was used to manage retrieved citations.

## Mechanisms and interactions of PGRs in rice

**Figure 2:**
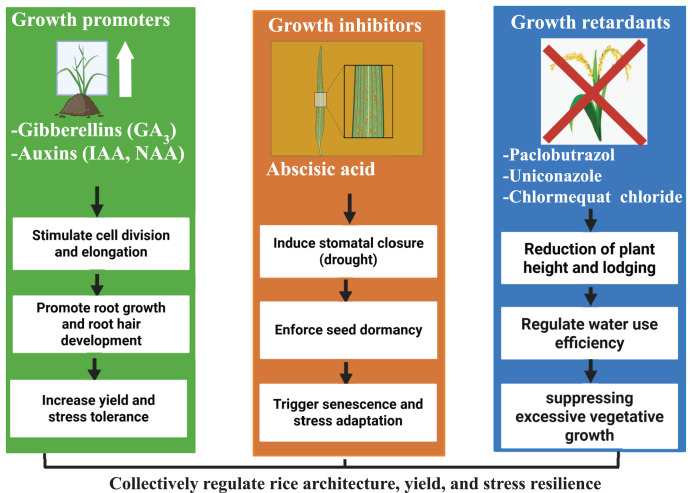
A hypothetical model of PGR functional groups and their primary functions in rice. Functional categorization of major plant growth regulators (PGRs) in rice. PGRs are classified into three groups based on their primary physiological effects. Growth promoters (left) stimulate developmental processes such as germination and cell elongation. Growth inhibitors (center) suppress growth, often as an adaptive response to environmental stresses. Growth retardants (right) slow vegetative growth to prevent lodging and improve resource allocation. This classification provides a framework for understanding targeted PGR applications. Created in BioRender. https://BioRender.com/wxvv7m3.

PGRs have diverse effects on rice *via* the modulation of distinct biochemical pathways and direct gene regulation in dynamic crosstalk networks to shape the growth, architecture, and abiotic stress tolerance of rice. These regulators are classified as growth promoters (auxins, GAs, cytokinins), inhibitors (ABA, ethylene), and retardants (paclobutrazol, uniconazole, chlormequat chloride), and can regulate rice ontogeny of germination to grain filling (Bai et al., 2021; Mishra et al., 2024; Sosnowski, Truba & Vasileva, 2023). The synergistic and antagonistic interactions between these PGRs, as demonstrated in Fig. 2, allow plant architecture and yield to be precisely adjusted across different environments (Kalkan et al., 2025).

### Core mechanisms by functional class

#### Growth promoters

Cytokinins (kinetin, 6-BA) and auxins (IAA, NAA, IBA, GA) mediate cell division, cell expansion, and cell differentiation, which are essential in vegetative vigor and reproductive success (Bisht et al., 2018; Hafeez et al., 2024; Jameson & Song, 2016). GA_3_ interacts with the soluble receptor GID1 and results in the ubiquitination and subsequent degradation of DELLA protein mediated by *GID2*, and derepresses growth genes, such as *GA15b*, to loosen the cell wall, extend internode, and exert panicle (Bilal et al., 2023; Spielmeyer, Ellis & Chandler, 2002). This increases α-amylase within aleurone layers to release reserves that are used to carry out germination and seedling development (Ueguchi-Tanaka, 2023). Auxins stimulate the TIR1/AFB receptors, causing the degradation of Aux/IAA repressors and liberation of ARF transcription factors to promote root and shoot growth (Lin et al., 2021; Majda & Robert, 2018; Soomro, Soomro & Mazari, 2020). Through Cytokinins, AHK receptors phosphorylate AHP histidine phosphotransmitters and ARR response regulators, which promote, respectively, tillering (*via* OsWOX11/OsERF3), chlorophyll retention, and delay senescence by suppressing OsNAP (Sammi Reddy et al., 2025; Tanaka, Yamauchi & Tsuda, 2023; Zha et al., 2022). Their application improves root proliferation before transplant and increases the number and yield of white roots by 10–15% (Tang et al., 2024).

#### Growth inhibitors

ABA and ethylene are adapted to endure drought, salinity, or flooding. PYR/PYL/RCAR receptors of ABA stimulate SnRK2 kinases by inhibiting PP2C phosphatases, allowing ABI5/bZIP factors and SLAC1 channels to be phosphorylated and close stomata rapidly (by 50–70 per cent) to reduce transpiration (Bhatnagar et al., 2020; Hirayama & Umezawa, 2010; Soma et al., 2023). In the downstream, ABA increases *OsNCED1* to biosynthesize feedback and decreases GA through OsAP2-39 to save energy (Shu, Zhou & Yang, 2018). Ethylene through ETR1/ERS receptors releases EIN2/EIN3, which triggers senescence (chlorophyll breakdown), upregulates SUB1A-1 to induce quiescence during submergence, and strengthens culms against lodging and stimulates adventitious roots (Hussain et al., 2020; Ju et al., 2022; Qin et al., 2024). Crosstalk between ethylene-auxin through OsEIL1 enhances MHZ10/REIN7, which improves root plasticity under salt stress conditions (Ma et al., 2024; Mickelbart, Hasegawa & Bailey-Serres, 2015).

#### Growth retardants

Triazoles (paclobutrazol, uniconazole) inhibit ent-kaurene oxidase and early GA pathways, reducing bioactive GA concentrations and DELLA repression to reduce internode length and prevent lodging (Shah et al., 2019; Wu et al., 2022; Zhou et al., 2025). They augment the accumulation of lignin and cellulose on culm walls, redirect assimilates to grains, and low doses at 200 mg/L foliar spray improve root, photosynthesis, and salt tolerance by curbing MDA and H_2_O_2_ as well as enhancing SOD and POD (Liu et al., 2024a). Soil drench application of paclobutrazol at the tillering stage increases yield under stress conditions (Setiawan et al., 2024; Tousi, Esfahani & Hosseini Chaleshtori, 2025). Applications, dosages, and outcomes are summarized in Table 2 to aid field protocols.

**Table 2:** Key plant growth regulators in rice: functions and prospects.

PGR name	Primary role in rice	Application time/method	Effects	Reference
Auxins (IAA, NAA)	Rooting, cell elongation, and tillering	Seed soaking and foliar spray	Stimulate root/shoot growth, increase the number of tillers, and improve nutrient uptake.	Mohapatra et al. (2025), Soomro, Soomro & Mazari (2020)
GA_3_	Coleoptile and mesocotyle elongation, seed germination, breaking dormancy	Foliar spray at booting/heading	Early maturity, improved spikelet fertility, breaking of seed dormancy	Liao et al. (2023), Wang et al. (2021)
Cytokinins	Promotes cell division, delayed leaf senescence, spikelet differentiation	Foliar spray at the tillering/booting stage	Increases effective tillers and spikelets, grain set enhancement	Sammi Reddy et al. (2025), Wu et al. (2019)
ABA	Regulates stomatal closure, drought stress, and seed maturation	Foliar application at late growth	Improves stress resistance, promotes grain filling, and influences maturity	Fahad et al. (2016), Saradadevi, Palta & Siddique (2017)
Ethylene	Modulates grain filling, ripening, and senescence	Not typically exogenous in the field	Balances grain filling, involved in stress signaling	Fahad et al. (2016), Panozzo et al. (2025)
Brassinolide	Provides root/shoot growth, photosynthesis, and stress tolerance	Seed soaking, seedling/vegetative spray	Increases seed germination, chlorophyll synthesis, and better tolerance to stress	Fahad et al. (2016), Islam et al. (2024)
Salicylic acid	It induces a defense response, involved in resistance to biotic/abiotic stress.	Foliar spray at various stages	Enhances tolerance against stress or pathogen attack and modulates the excess of oxidative damage	Song et al. (2023), Wang et al. (2024)
Jasmonic acid	Elicits defence mechanisms, alleviates abiotic stress	Foliar spray during the reproductive phase	enhancing thermotolerance, regulating spikelet development	Rehman et al. (2023), Wu et al. (2019)

**Note:**

PGR, Plant growth regulator; GA_3_, gibberellic acid; IAA, Indole-3-acetic acid; NAA, naphthalene acetic acid; ABA, abscisic acid; concentrations and application timings are generalized from the cited literature; specific protocols may vary by genotype and environmental conditions. This indicates that a standard field application method for exogenous ethylene is not commonly specified in the cited studies.

The derivatives of cyclohexanedione are contemporary retardants that are highly active and rapidly decompose (Mykhalska, Makoveychuk & Schwartau, 2021). Trinexapac-ethyl (TE, 75-300 g ai ha-1 at 6th-7th leaf), which is a GA20ox inhibitor, decreases height by 10–20 percent, improves head rice yield (5–15 percent), and reduces lodging without a yield penalty (Corbin et al., 2016; Sezer et al., 2016). Prohexadione calcium (Pro-Ca, 100 mg L^−1^ foliar) also reduces the length of internodes, improves the lodging resistance, and enhances photosynthesis and antioxidants under the stress condition (Huang et al., 2023; Mei et al., 2025; Ningbo Inno Pharmchem Co., Ltd, 2025). Compared to triazoles, cyclohexanediones have shorter soil half-lives, minimizing carryover risks, as referenced in Table 2 above.

### Hormonal crosstalk and synergy

Phytohormonal regulation in rice operates through complex and dynamic crosstalk networks involving antagonistic and synergistic interactions among major hormones such as ABA, GA, auxin, and cytokinins (Aerts, Pereira Mendes & Van Wees, 2021; Altmann et al., 2020). ABA-GA antagonism can be maintained at the level of homeostasis. ABA induces *OsAP2-39* to inhibit *GA20ox*/*GA3ox* and activate *EUI* catabolism, thereby reducing the GA1/ABA ratio and targeting dormancy and dwarfing. GA then responds through DELLA feedback (Du et al., 2015; Shu, Zhou & Yang, 2018; Shuai et al., 2017). Recent ABA-5-HT crosstalk inhibits ABI5 phosphorylation *via* SAPK2, thereby enhancing serotonin without penalty at the expense of yield (Cui et al., 2025).

Resistance to stress conditions is enhanced by synergies. The interaction between ethylene and GA facilitates the growth of coleoptile and adventitious roots through *SUB1A* and *SNORKEL1/2* upregulation and auxin accumulation (*MHZ10/REIN7*), and prevents flooding conditions, which is blocked by ethylene (Nagai & Ashikari, 2023; Qiao et al., 2024). Auxin-cytotokinin antagonism through the ROP GTPases enhances levels of the shoot-root ratio, cytokinins (ARR5/6) antagonize PIN auxin efflux to maintain tillering dominance, restrained by the strigolactones (Mao et al., 2020). Cytokinin-auxin antagonize the effect of ABA in stomatal closure through ethylene regulation (Tanaka et al., 2006).

Recent discoveries indicate that BR integration increases architecture-related auxin and GA responses, whereas ABA and ethylene crosstalk improve salinity tolerance through K^+^/Na^+^ homeostasis (Altaf et al., 2025). These are enhanced by synthetic PGRs through specific analogs.

**Figure 3:**
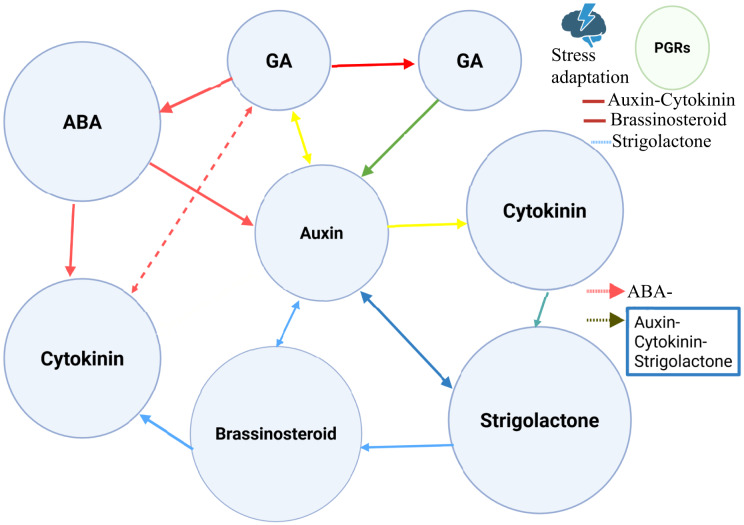
Network representation of the most significant PGR interactions and their targets. Core crosstalk network among major phytohormones in rice. Key synergistic, antagonistic, and regulatory interactions between plant growth regulators (PGRs). Line colors and styles denote the nature of the relationship: red blunted lines for antagonism, green arrows for synergy, and blue lines for complex, context-dependent antagonism. This interconnected network allows rice plants to integrate multiple developmental and environmental signals, fine-tuning responses for optimal growth and stress adaptation. Key outcomes of these interactions are annotated. Created in BioRender. https://BioRender.com/img8694.

This crosstalk (Fig. 3) gives plasticity, ABA-GA seeds resistance to precocity, ethylene-GA escapes submergence, auxin-cytotokinin-BR creates ideal architecture, and there are no single-hormone constraints (Fahad et al., 2016; Liu & Hou, 2018). The combined application of paclobutrazol and GA₃ enhances yield gains under stress and is ideal in precision agriculture (Maheshwari et al., 2022).

## Effects of PGRs on rice growth and development

PGR usage is aimed at enhancing the growth of rice at particular stages of its development, architecture, formation of the yield, and other stress resistance mechanisms, directly based on the mechanisms and crosstalk described in ‘Mechanisms and Interactions of PGRs in Rice’.

### Early growth stages

Plant establishment and yield potential depend on seedling vigor, which is achieved through GA3 and ethylene, which break dormancy by inducing α-amylase and mobilizing reserves (Del Rosario & Valdez, 2025; Savaedi et al., 2019; Shukla & Pathak, 2025). In saline conditions, the application of GA_3_ at 50–100 ppm priming of the seeds can reinstate germination through balancing decreased endogenous GA, increased water imbibition, metabolism, and emergence rate (Larson et al., 2023; Singh & Kandel, 2025; Wang et al., 2021). GA_3_ and ethephon have a synergistic effect of augmenting antioxidant preparedness with ethephon priming, which improves starch hydrolysis in low moisture conditions to attain strong root architecture and oxidative shield (Watanabe et al., 2018; Zhang et al., 2024a).

Application of auxins in the form of NAA and IBA at 25 ppm promotes adventitious roots essential to anchorage, which are amplified by cycocel and mepiquat at 10 ppm combinations, resulting in profuse roots (Mohapatra et al., 2025). The SOD and CAT are increased by the SA foliar sprays at the two-leaf stage, and MDA and H_2_O_2_ are reduced to maintain the chlorophyll and vigor during stressed conditions (Shan et al., 2024; Zafar et al., 2021). A balance between root-shoot growth is reached through GA and cytokinin synergy to have uniform stands (Aloni et al., 2006; Dong et al., 2023).

### Tillering and stem elongation

Tillering is an antagonistic action of cytokinins and inhibition of the axillary buds by strigolactone (Rasheed et al., 2023; Wen et al., 2024). High concentrations of auxin in the main stem prevent auxin transport in axillary buds, causing dormancy; the increased potency of NAA, compared to IAA, supports strigolactone-mediated inhibition (Chabikwa, Brewer & Beveridge, 2019; Jia et al., 2024; Noor et al., 2020). The cytokinin has an antagonistic effect through *OsWOX11* and *OsERF3*, which maximizes productive tiller yield (Basuchaudhuri, 2024).

GA_3_ mediates the elongation of the stem that supports the division of internodes in dwarf plants, contributes to panicle exertion, seed sets, and energy provision by α-amylase (Gao & Chu, 2020; Rakesh et al., 2024; Spielmeyer, Ellis & Chandler, 2002). Lodging is induced by large amounts of nitrogen and strong winds, but the use of retardants counteracts this damage (Niu et al., 2021). The inhibition of ent-kaurene oxidase by paclobutrazol and chlormequat shortens internode length. Also, it thickens culm and shifts the assimilates to panicles, provided the dosage used is precise and does not lead to yield penalties (Table 3) (Kamran et al., 2018; Ren et al., 2022; Setiawan et al., 2024).

**Table 3:** Management of PGR application in different growth stages and situations in rice.

Growth stage	Target challenge/goal	Key PGR(s)	Application strategy	Recommended formulation & practical considerations	Expected outcome	Reference
Germination	Uniform seedling stand, rapid establishment	Gibberellins, Auxins	Seed pre-soaking, soil drench	GA_3_ (20–50 ppm) for seed priming to break dormancy; IBA (10–25 ppm) to promote root initiation. Application can be useful especially in stressful environment like salinity and suboptimal temperature.	Enhances germination, increases vigor, and promotes root initiation.	Del Rosario & Valdez (2025), Du et al. (2022), Soomro, Soomro & Mazari (2020)
Early vegetative	Tillering, initiation of tiller buds	Cytokinins, Auxins	Foliar spray at active tillering	Cytokinins like 6-BA (30–50 ppm) as a foliar spray at the active tillering stage. Application should be targeted at high-yielding but low-tillering genotypes to maximize effect.	Boosts tiller number, facilitates a robust shoot system.	Chatterjee et al. (2025), Wu et al. (2019)
Stem elongation	Lodging prevention	Prohexadione-calcium, Paclobutrazol	Foliar spray at the internode elongation phase or jointing	Paclobutrazol (100–300 ppm) as a foliar spray at the jointing stage, especially in tall, lodging-susceptible varieties grown under high nitrogen fertilization. Not recommended for dwarf varieties.	Reduces stem elongation, improves stem strength, and mitigates lodging.	Kamran et al. (2018), Ningbo Inno Pharmchem Co., Ltd (2025)
Reproductive (Booting/Heading)	Increased grain set, pollen viability	Cytokinins, Gibberellins, Brassinolide	Foliar spray at booting or pre-heading	A combination of 6-BA (30 ppm) and GA_3_ (10–20 ppm) at booting can improve panicle exertion and spikelet fertility. Dosage must be carefully calibrated to avoid excessive stem elongation.	Increases spikelet differentiation and grain number, improves yield.	Aryal & Alferez (2025), Pantoja-Benavides, Garces-Varon & Restrepo-Díaz (2022)
Grain filling	Enhanced grain filling, stress tolerance	Abscisic acid, Brassinolide, Jasmonic acid	Foliar spray during early grain-filling	Foliar application of ABA (10–20 uM) or Brassinolide (0.1–0.5 ppm) at the beginning of the grain filling stage can increase the amount of starch stored in the grains, particularly in low quality spikelets and under terminal drought stress.	Promotes grain filling, counters high temperature and drought stress.	Wang et al. (2025), Zulfiqar et al. (2024)

**Note:**

PGR, Plant growth regulator. The recommended application strategies and expected outcomes are based on research under controlled or specific field conditions. Optimal dosage and timing should be calibrated for local varieties, soil properties, and prevailing climatic factors to achieve the stated goals and avoid phytotoxicity.

### Flowering and panicle initiation

Floral determinacy of meristem and stress responses during vegetative and reproductive transition are dependent on cytokinin-ethylene balance (Jiang et al., 2018; Parida et al., 2022; Ye et al., 2019). Exogenous application of 6-benzyladenine activates the shoot apical meristem (SAM), promotes primary and secondary branch proliferation, and upregulates *OsMADS1* and *OsMADS14*, which in turn regulate panicle architecture and floral organ identity even in the developmental delays caused by abiotic stress (Miao et al., 2022; Molla, 2022; Wei et al., 2024). The cytokinin/auxin interaction is what maintains apical dominance, as well as inhibits branching, and is a common occurrence across species, including in apple *via MdTFL1* and *AFL1* (Tan et al., 2019).

### Promotion of grain filling and yield

Through grain filling, the sink strength is controlled by the dynamics of sucrose and starch flow between the source and sink (Chougule & Rawat, 2024; Durbha et al., 2024). ABA increases the rate of starch and protein through synthases upregulation in inferior spikelets but poses a risk of senescence when applied in excess (Zhu et al., 2011). The activation of OsBZR1 by brassinosteroid signaling stimulates endosperm growth, improves the partitioning of hexoses and amino acids, enlarges the grain size, and increases the expression of heat-shock proteins during heat stress and K^+^/Na^+^ homeostasis under salt stress (Manghwar et al., 2022; Percio et al., 2025; Zeng et al., 2024). OsNAP senescence is suppressed by cytokinins (6-BA), which maintain photosynthesis, chlorophyll, and enhance NUE (Abualia, Riegler & Benkova, 2023; Glanz-Idan et al., 2022).

**Figure 4:**
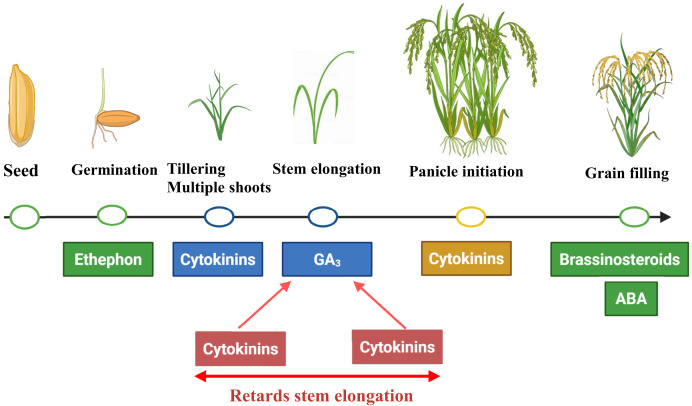
Effect of PGRs on primary growth stages, from germination to grain filling. Stage-specific influence of major plant growth regulators (PGRs) during the rice life cycle. The schematic timeline illustrates key developmental stages and the primary PGRs that exert critical influence at each phase. Green highlights generally growth-promoting effects, while blue indicates growth-retarding applications used agronomically. The complex role of hormones, such as ABA, during grain filling is noted. This stage-specific understanding is vital for timing PGR applications to maximize their efficacy on growth, architecture, and ultimate yield. Created in BioRender. https://BioRender.com/1nwuwws.

Jasmonates and polyamines (spermidine) can increase the grain filling and stress tolerance through the process of ROS quenching (Berahim et al., 2021; Rehman et al., 2023). Interactions between PGRs show that decreased ABA and GA_3_ enhanced grain filling; combinations of GA_3_, PBZ, and 6-BA at heading increased the yield in Peizataifeng and Huayou 86, BRs, MeJA, and triazoles enhanced salt-stress tolerance in IR-64, GA_3_ and SA promoted salt-stress yield in CSR-36 and CSR-43, and 6-BA inhibited senescence (Chen et al., 2020; Fahad et al., 2016; Pan et al., 2022; Pratap et al., 2018). The effects of these PGRs on the germination of seeds to grain filling, affecting their potential agronomic use, are shown schematically in Fig. 4, depending on the stage. It is important to mention that the application of PGRs is a strategic measure and not a fixed protocol; the best results are obtained by focusing on certain developmental limitations, such as using retardants to control lodging or cytokinins to initiate panicle instead of hormones at all stages.

Strategic adjustment of the hormonal environment *via* rational dosages, timing, and hormone combinations can efficiently enhance grain filling, nutrient translocation, and spikelet fertility (Panozzo et al., 2025). The grain yield of rice has been proven to be enhanced by several PGRs, as indicated by various field and pot studies (Table 4). These results not only demonstrate the general benefits of PGRs such as GA_3_, 6-BA, and SA consistently observed in this study, but also point to future mitigation potential by exploring synergistic combinations, such as brassinosteroids with methyl jasmonate or beyond. As global climate change continues and we strive for food security, at least by maintaining rice productivity, precise use of PGR is likely to play an important role in counteracting erratic changes in climatic parameters while compensating for unverifiable resource loss when moving cultivation from the field to a controlled environment.

**Table 4:** Effects of PGR treatments on rice grain yield.

PGR treatment	Concentration	Cultivar	Grain yield (control)	Grain yield (treated)	Percentage change *vs*. control	Experimental notes	Reference
GA_3_	20 ppm	Peizataifeng	28.32 g/plant	31.68 g/plant	+11.9%	**Pot study;** Foliar at panicle initiation	Pan et al. (2013)
GA_3_	20 ppm	Huayou86	33.12 g/plant	36.16 g/plant	+9.2%	**Pot study;** Foliar at panicle initiation	Pan et al. (2013)
6-BA	30 ppm	Huayou86	33.12 g/plant	34.48 g/plant	+4.1%	**Pot study;** Foliar at panicle initiation	Pan et al. (2013)
GA_3_	200 ppm	CSR36	7.32 g/plant	9.21 g/plant	+25.8%	**Pot study under salinity stress;** Seed priming	Pratap et al. (2018)
SA	200 ppm	CSR43	8.11 g/plant	9.12 g/plant	+12.5%	**Pot study under salinity stress;** Foliar	Pratap et al. (2018)
Br + Tr + MeJA	4/0.55/1.8 ppm	IR-64	13.9 g/plant	19.5 g/plant	+40.3%	**Pot study;** Foliar, stress mitigation	Fahad et al. (2016)
Prohexadione-calcium	35 g/ha	Hybrid rice	7.41 t/ha	8.58 t/ha	+15.8%	**Field trial;** Foliar, jointing stage	Liao et al. (2023)
Indole-3-Acetic Acid (IAA)	50 ppm	Hybrid rice	41.9 g/plant	48.4 g/plant	+15.5%	**Pot study;** Foliar, tillering to maturity	Soomro, Soomro & Mazari (2020)
Indole-3-Butyric Acid (IBA)	25 ppm	Hybrid rice	41.9 g/plant	46.3 g/plant	+10.5%	**Pot study;** Foliar, tillering to anthesis	Shah et al. (2023)

**Note:**

PGR, Plant growth regulator; GA_3_, gibberellic acid; 6-BA, 6-benzylaminopurine; SA, salicylic acid; Br, brassinolide; Tr, triazole compound; MeJA, methyl jasmonate; IAA, indole-3-acetic acid; IBA, indole-3-butyric acid; t/ha, tonnes per hectare. Percentage change is calculated as [(Treated – Control)/Control] × 100. Yield values are as reported in the respective references. The experimental conditions (pot/field, stress imposition) significantly influence the magnitude of response.

## Economic, regulatory, and practical considerations for PGR adoption

### Economic feasibility and field-scale efficacy

Despite studies on the agronomic potential of PGRs conducted in controlled settings, their large-scale implementation requires demonstrating economic feasibility in real-world agro-phytocenoses. One of the most important gaps in the existing literature is the lack of solid on-farm trial data, which incorporates a complete cost-benefit analysis. As stated in Table 3, some of the PGR treatments, such as the combination of brassinosteroid-triazole reported by Fahad et al. (2018), demonstrated an encouraging 40 percent yield improvement during stress, but these findings need to be confirmed in a variety of farming systems and scales. It is not economically viable; it hinges on whether the monetary benefits of higher yield or loss mitigation, such as lodging prevention, obviously exceed the expenditures of the PGR product, application equipment, and labor. The stability of PGR in the field, the requirement of exact application timing, and specific reactionary response to certain genotypes provide the added complexity and risk, especially to smallholder farmers who may find the initial investment in the technology to be prohibitive. It is recommended that future research be carried out through long-term, multi-location field experiments to obtain valid information on investment returns.

PGRs are economically viable and worth targeted implementation for smallholders when applied strategically. Field experiments have shown a benefit-cost ratio of 2–5:1. For instance, paclobutrazol at $10–20/ha prevents lodging, yielding 15–30% yield gains and netting $100–300/ha across Asian trials (Bakhsh et al., 2012; Jadhav et al., 2020; Wu et al., 2024). For farmers with limited resources, generic formulations at low doses (less than $10/ha), government subsidies (as in China/India), and on-farm demonstration plots achieve 20–50% adoption rates by alleviating timing and genotype risks through farmer training. While there are still challenges in global application, tactical piloting of high-value rice systems can yield high returns, making PGRs accessible to subsistence farms (Table 5).

**Table 5:** Economic viability of PGRs under different farm scenarios, showing net yield gains, benefit: cost ratios, and smallholder viability.

Scenario	Cost/ha (USD)	Yield gain	Net benefit/ha (USD)	Benefit-cost ratio	Smallholder viable?	References
Commercial farms	15–25	20–40%	+200–500	5–10:1	Yes	Bakhsh et al. (2012), Singha et al. (2025)
Smallholder (subsidized)	<10	15–30%	+100–200	3–5:1	Yes, *via* demonstration	Ouma et al. (2024), Woittiez et al. (2018)
Smallholder (no subsidy)	10–30	10–25%	+50–100	2–3:1	Conditional	Yadav et al. (2023)

**Note:**

Values based on maize baseline yields of 2–5 t/ha; subsidized scenarios include 50% input cost subsidy and extension services.

### Regulatory frameworks and environmental safety

Synthetic PGRs are regulated for use in agriculture to ensure environmental and consumer safety. Regulations, including those devised by the Environmental Protection Agency, ought to include Maximum Residue Limits (MRLs) of PGRs in food products (Zhang, 2025). Nonetheless, the possibility of residue concentration in rice grains, as noted in ‘Technical and Environmental Risks’, is a relatively underexplored field. Setting up of MRLs and the degradation Kinetics of these compounds are crucial steps to be taken. In addition, the continuous use of persistent PGRs, such as paclobutrazol, which may persist in the soil up to several months, requires a particular delivery of these components with caution so as not to induce carryover effects on the new crop and pollute the water systems (Zhao et al., 2021). Such a regulatory environment underscores the need to design and strongly embrace readily biodegradable and environmentally friendly PGR formulations to reduce environmental effects.

### Towards reducing chemical inputs: integration and accessibility

One of the critical approaches to sustainable crop production is the minimization of total chemical inputs (Khursheed et al., 2025). In this regard, PGRs should not be considered as mere additions but as precision tools in Crop Management strategies. It aims at employing the PGRs to maximize the efficiency of other inputs, like the enhancement of nitrogen use efficiency (NUE) to minimize fertilizer requirement or prevent losses, hence making the resource input more productive. A key obstacle is the economic accessibility of farmers, particularly for smallholders. The measures to enhance accessibility should cover the establishment of generic formulations that are affordable, governmental subsidies, and education to farmers that can help them understand the circumstances under which PGR use is safe and economically feasible. The future of PGR application is smart, targeted use that has the greatest benefit but the least external input and environmental impact.

## Synergistic integration with agronomic practices

Integration of PGRs and appropriate agronomic management strategies is a powerful phenomenon to enhance rice yield and resource-use effectiveness, as well as to enhance sustainability (Saradadevi, Palta & Siddique, 2017). PGRs have been shown to have a synergistic potential of enhancing physiological performance and stress resistance and decreasing lodging and production loss in combination with optimal nutrient management, breeding programs, and precision agriculture (Liao et al., 2023; Mansha et al., 2025). Here, we emphasize the role of cytokinins in increasing NUE and discuss how combining them with MeJA, along with morpho-physiological approaches developed through advanced breeding strategies, can optimize these potential improvements in rice tolerance and yield stability.

### Nutrient use efficiency and broader plant nutrition interactions

Cytokinins improve nitrogen use efficiency by stimulating nitrogen uptake and translocation. Cytokinins increase the NUE by boosting nitrogen uptake and translocation, especially under stress conditions (Zhu et al., 2022). Cytokinins play a regulatory role in nitrogen uptake and assimilation by interacting with nitrogen signaling pathways and transporter activity, including nitrate transport systems in rice (Sakakibara, 2021). Application of cytokinin either alone or with other essential nutrients has been observed to speed up the nitrogen transport process in old basal leaves to young tissues and extend the senescence process alongside maintenance of canopy photosynthesis during stressful situations (Shah et al., 2023). The use of cytokinin in rice can boost the nitrogen content of the leaf, as well as the root (Zhu et al., 2022). Similarly, the combined application of cytokinin, ethylene, and auxin to rice plants led to an increase in the total nitrogen content of the leaves (Li et al., 2018). These results indicate that cytokinin-involved control of nitrogen metabolism is not only favorable for the adaptation of plant growth to low-nutrient or stress-prone conditions, but it also promotes the photosynthetic acclimation at the canopy level, ensuring the convenient use of assimilates in support of grain development.

Nonetheless, hormonal crosstalk may be complex as well, depending on the situation. For instance, MeJA treatment on rice seedlings is found to reduce root nitrogen uptake and root-shoot translocation of ^15^N, resulting in down-regulation of the metabolic processes responsible of nitrogen assimilation, Glutamine Synthetase and Nitrite Reductase enzymes (Hou et al., 2021). Interestingly, MeJA-treated shoots can transfer the available endogenous nitrogen from leaves to roots, indicating a compensatory shift in nitrogen remobilization rather than an absolute increase in assimilation (Wu et al., 2019). These results demonstrate that the fine-tuning of PGRs, such as Cytokinin and MeJA, in combination with a balanced nutrient status, can improve NUE and delay senescence in rice plants. In this model of synergy management, sustainable productivity and sustainability are maintained, and less fertilizer dependency and environmental pollution are reduced.

#### Broader nutrient interactions

PGRs react with non-nitrogenous macronutrients. GA_3_ and BRs enhance the phosphorus (P) uptake during low-P stress by increasing the expression of OsPHT1 transporters, which improves root architecture (Aryal & Alferez, 2025; Usmani et al., 2025; Wang et al., 2024). Salinity-induced K^+^ homeostasis is mediated by ABA-ethylene crosstalk, with paclobutrazol stabilizing K^+^/Na^+^ ratios through OsAKT1 upregulation (Mohsin et al., 2019; Singh et al., 2022). Prohexadione calcium (Pro-Ca) enhances Mg acquisition under salt stress, alleviating Mg deficiencies that limit photosynthesis (Deng et al., 2024). The exogenous application of cytokinins and N enhances P/K remobilization to grains, and triazoles divert assimilates *via* imbalanced fertilization (Li et al., 2022). Such relations underscore the importance of PGRs in enhancing nutrient efficiency in NPK spectra, especially under abiotic stress.

### Breeding strategy

The recent advancement in transgenic technology and plant breeding revived the hope of genetic traits improvement coupled with PGR hormonal regulation. The current breeding technologies can modify the endogenous hormonal pathways to produce rice varieties that are more tolerant to stress, more productive, and efficient in resource utilization. Over the recent years, it has been demonstrated that gene editing technologies, such as CRISPR-Cas9, can be used to produce rice mutants with modified hormone metabolism and signaling (Luo & Liu, 2025). For instance, the transcription of the gene *OsGA20ox2* is suppressed by RNAi, which causes a decrease in plant height due to reduced GA production, resulting in lodging resistance and an increase in yield (Qiao & Zhao, 2011). Furthermore, the introduction of rice resistant to herbicides would also allow the application of broad-spectrum herbicides, which are required to control weeds and manage the field more effectively (Pervaiz et al., 2024). However, these benefits should also be weighed against the potential environmental and economic consequences. High dosage and frequent application of PGRs destroy soil microbes, disrupt plant-soil interactions, and affect plant physiological function (Strydhorst, Hall & Perrott, 2018; Wang, Chi & Song, 2024). It is worth noting that we are still at the very early stage of endorsing the use of nano-particles in field conditions. Hence, it is now necessary to conduct ideal dose calibration and long-term ecological evaluation, which are required for sustainability (Faizan & Hayat, 2024). In conclusion, the integration of biotechnological breeding advances with well-planned PGR use emerges as a promising frontier in rice intensification and climate-smart agriculture. Further studies of gene-hormone interactions, PGR formulation modification, and ecological safety will be essential to maximize their utilization in sustainable rice production systems.

## Challenges and risks

Although PGRs offer numerous agronomic and physiological benefits for rice cropping, their incorrect or excessive application poses several technical, environmental, and socio-economic risks. These problems are primarily related to phytotoxicity, environmental degradation, food safety concerns, and economic challenges that hinder the sustainable utilization of these agents (Hasanuzzaman et al., 2020; Le et al., 2020).

### Technical and environmental risks

The continual and unrestrained application of PGRs might cause phytotoxicity and hormonal disturbance in rice, as evidenced by researchers (Nita et al., 2026). For example, excessive accumulation of auxins disrupts the endogenous hormone balance, resulting in dwarfism, panicle malformation, and reproductive defects (Jin et al., 2016; Vanneste, Pei & Friml, 2025). Likewise, the overuse of paclobutrazol limits photosynthesis and root elongation, thereby hindering biomass accumulation and yield capacity (Huang et al., 2019). Moreover, the residues of PGRs may remain in soil and plants, resulting in environmental pollution and affecting food safety, such as food poisoning, and inhibiting their growth (Goel et al., 2024).

There is a knowledge gap on the possible translocation and build-up of synthetic PGRs in rice grains. Whereas endogenous hormones are highly regulated, the response of exogenously administered synthetic analogs is less well-known. Some PGRs do not degrade easily and are systemic; they may be translocated to developing seeds, which poses a risk to food safety and unwanted dietary exposure. The correlation between time of application, dosage, and the amount of final residue in the grains requires careful research. The relationship between application timing, dosage, and final residue levels in harvested grains needs thorough investigation. Future studies should focus on investigations involving the application of better analytical methods in order to monitor the absorption, metabolism, and ultimate deposition of the regularly employed PGRs within the various tissues of rice, especially the edible grains. Determining the maximum residue limits (MRLs) and the degradation dynamics of these substances during the grain-filling stage is also a crucial step to make sure that the area is safe and can regulate the policies regarding the safe usage of the PGRs in the rice production process.

The instability of certain PGRs in light and temperature, in addition to the field performance, may complicate their penetration upon degradation with resultant inconsistent activity (Gao et al., 2025).

### Scalability and risks associated with large-scale application

PGRs are promising, but the effect is not consistent across all crops. The differences are caused by genotypic specificity, environmental differences, and application nuances. For example, GAs often promote stem elongation but can cause lodging in tall varieties, whereas they have minimal effects in dwarf genotypes with altered GA pathways (Hussain, 2025). Moreover, the effects of the same PGR can have opposing effects depending on dosage. For instance, low doses of cytokinins stimulate tillering, while high doses suppress root growth. The major constraint of the existing literature, as indicated in Table 4 of this review, is that most data is available in pot or small-field research, and there is a relative lack of validation on a hectare scale. The consequences of this lack of data directly contribute to the danger of unpredictability, because the assumptions made as a result of the small-scale experiments might not be translated to the heterogeneous conditions of the actual fields.

PGR use is not economically feasible, and the threat is the possibility of a negative return on investment. Cost-benefit imbalance occurs when the expenses incurred in the purchase of the PGR product and its usage exceed the economic benefits of either an increase or a loss reduction in yield (Ouma et al., 2024; Yadav et al., 2023). The initial capital and the technical expertise needed to invest in the smallholder farmers can be daunting, and worsen the chances of socio-economic disparity. Furthermore, the instability of PGRs in field conditions, the high requirements of special sprayers to cover the entire area, and the exact time of PGR application all contribute to the risk of failure in the application process and uneven effectiveness. Thus, on-farm trials over a long period of time in various agro-ecologies should be of primary concern in future studies to produce credible cost-benefit information capable of measuring these risks.

### Long-term ecological consequences and soil health

Although the agronomic advantages of the PGRs are obvious, a detailed sustainability evaluation should take into account their long-term environmental destiny and soil health effects. The degradation rates of synthetic PGRs in rice field soils are influenced by multiple factors, including their chemical structure, application method, soil pH, organic matter content, microbial activity, and water management practices. As an example, it was reported that paclobutrazol, a retardant of the persistent type (a triazole), has a soil half-life of weeks up to several months, which is a threat to carryover effects to the subsequent crops and potential leaching into the water systems (Wu et al., 2013). Much less stable compounds, such as GA_3_, on the other hand, break down fairly easily by microbial and photochemical means, reducing long-term accumulated effects.

Excessive or continued use of synthetic PGRs may have serious ecological effects in the long-term, and these are mainly caused by interference with soil microflora. The microbes play a crucial role in the cycling of nutrients, decomposition of organic matter, and soil fertility. The microbes play a crucial role in the cycling of nutrients, decomposition of organic matter, and soil fertility. Some of the PGRs or their metabolites may serve as general biocides or change the pH of the soil, causing a decrease in the diversity and biomass of microorganisms (Gao et al., 2025). Such interference may be detrimental to mycorrhizal relationships, which are important to nutrient uptake by plants, and may decrease the population of beneficial bacteria engaged in nitrogen fixation and phosphate solubilization. The anaerobic conditions that prevail in flooded paddy fields may dramatically modify the degradation processes, which may give rise to the generation of transformation products whose complete ecological effects are yet to be fully comprehended (Moharana et al., 2025). Such transformations in the soil biome may lead to a vicious cycle whereby the soil loses its natural ability to sustain plant growth and relies more on the use of chemicals. Moreover, the possibility of bioaccumulation of the persistent PGRs in the food chain and subsequent effects on the non-target organisms, like aquatic life in the nearby water bodies, is another environmental risk that needs further research (Ferreira & Mendes, 2021). Therefore, formulations of PGRs that can be readily biodegraded and which are preferentially used are essential to reducing environmental footprints, as well as the careful timing and dosage of application. Future studies should put more emphasis on ecotoxicological investigations and life-cycle evaluations in order to make sure that the PGR strategies are indeed consistent with the concept of sustainable intensification of agriculture to protect the health of soils, water, and the general environment integrity.

### Adoption barriers: economic constraints and implementation risks

Economic constraints and social barriers increase the physical and environmental risks associated with PGR application. High cost and specifications of application limit the access of PGR products among smallholder and subsistence farmers, posing a threat of inequality of technological access and, consequently, the widening of the gap between resource-endowed and disadvantaged farms (Lokhande et al., 2025; Zhumanova et al., 2024).

Moreover, the absence of technology access, awareness of the farmers, and support on extension are all factors, leading to a possibility of a knowledge gap and misuse (Touch et al., 2024). This may cause an optimum outcome, wastage of resources, or even phytotoxicity of crops. Although PGRs have been known to increase yield and tolerance to stress, they are still not fully utilized due to economic imbalances and managerial inefficiencies, and there is a risk of not fully utilizing the potential of PGRs to boost food security.

In the context of PGRs’ safe and efficient use in rice culture, one direction of future research should be to create biodegradable and environmentally-friendly PGR formulations, and also to conduct awareness creation programs to educate farmers on the methods of proper application. These are technical and socio-economic constraints that should be mitigated. These barriers can be overcome by applying PGRs in a targeted manner, using low-cost generics and demonstration plots, as detailed in Table 5, and providing cost-benefit ratios of 2–5:1 to smallholders.

## Emerging technologies and prospects

Progress in these technologies, such as biotechnology, nanotechnology, and digital agriculture, has been changing the future role of PGRs in rice crop applications. With considerable attention being focused on sustainable intensification and climate-smart agriculture (Hashim et al., 2024), these new developments aim to optimize delivery efficiency, enhance hormonal control accuracy, and minimize environmental footprints. In fact, recent research has shown that PGR-containing nanoformulations enhance their bioavailability, stability, and uptake efficiency, with the potential to achieve targeted delivery and release (Kah & Hofmann, 2014; Liu et al., 2024b; Petrovic, Bita & Barbinta-Patrascu, 2024).

**Figure 5:**
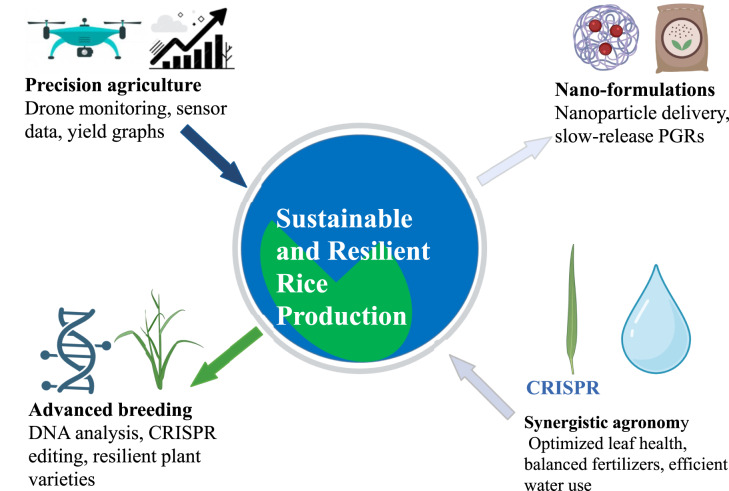
Utilization of PGR in conjunction with modern agriculture for sustainable rice production. Synergistic integration of PGRs with emerging technologies for the future of rice cultivation. The future of PGR use lies in its integration with other technological pillars. Precision agriculture ensures targeted application. Nano-formulations improve delivery efficiency and reduce environmental impact. Advanced breeding creates ideal plant architectures and stress-resilient genotypes. Synergistic agronomy combines PGRs with best management practices. Together, these interconnected approaches form a robust strategy for achieving sustainable intensification of rice production under changing climatic conditions. Created in BioRender. https://BioRender.com/pcvvk60.

These nanosystem carriers help protect PGR molecules in the environment, reducing dosage and application frequency (Tiwari et al., 2024). Moreover, the digital era has emerged with the full capabilities of remote sensing, data analytics, and artificial intelligence, which currently allow farmers to monitor crop responses, conduct physiological stress checks, and apply PGRs, optimizing timing and application rates (Markets and Markets, 2024; Swami et al., 2025). Using data to inform decision-making drives performance, enables more effective use of assets, and fosters better sustainability. The applications of these technologies present a model for precision-based PGR application in sustainable rice production, as shown in Fig. 5. At the genetic level, developments in CRISPR/Cas9 are allowing us to edit genes related to hormone production. For instance, targeted disruption of negative regulators in the ABA signaling pathway improves drought stress tolerance without a growth penalty (Choudry et al., 2024).

The combination of such technologies yields a more unified and flexible control system. The future of rice farming lies in nanotechnology, genetic engineering, and digital tools, as they facilitate the use of nanotech to deliver gene editing to maximize hormone concentration and digital tools to monitor crop performance. Such a combination will allow making future rice cultivation more responsive to evolving climatic conditions and efficient use of resources. Furthermore, biostimulants (substances or microorganisms that enhance nutrient uptake and improve plant health) are available, offering a more sustainable alternative to synthetic PGRs (Swami et al., 2025). They improve crop quality, minimize the application of chemical fertilizers, and enhance the efficient utilization of nutrients. Technology-driven PGR research and application may become a transformative element in achieving more sustainable and precise rice production systems. Future endeavors should focus on the commercial scalability of nanoformulated and biostimulant-based PGRs, the ethical oversight of genome-editing techniques, and the digital empowerment of farmers. In combination, these innovations can be substantially more productive and less ecologically and economically risky.

## Conclusion

Novel biotechnology, nanotechnology, and artificial intelligence-based precision agriculture technologies are transforming the future of PGR application, making it possible to regulate rice growth and productivity in a more targeted manner, while also reducing the environmental footprint. This article confirms that the use of PGRs at different developmental stages, for various genotypes, and within diverse agronomic contexts can significantly contribute to increasing yield, improving resilience to various stresses, and efficiently utilizing resources without a negative effect on the ecology.

The review highlights the need for scientists to investigate beyond the mechanistic research towards large-scale, on-farm experiments that can prove the PGR to smallholder farmers. It suggests the production of genotype-specific application regimes and environmentally sustainable and nano-formulated PGRs. A strategic framework is offered to agronomists on the most appropriate timing of applying PGRs to address production challenges that combine these strategies with precision agriculture tools to achieve sustainable intensification.

However, its application should be controlled or regulated because of the phytotoxic effects, long-term negative effects on soil health due to the retention of synthetic compounds like uniconazole, disturbance of the microbial community and nutrient cycling within the soil, environmental pollution, and socio-economic limitations in smallholder agricultural systems. As the world’s agriculture currently faces climate and nutritional crises, the integrated, judicious, and evidence-based use of emerging technologies alongside PGR management will be crucial for achieving food security and environmental sustainability. More efforts in interdisciplinary research, farmer education, and eco-friendly formulations will be needed to harness the full benefits of PGRs in future rice production.

Therefore, it is important to consider the application of PGRs as a precision tool to a more comprehensive approach in integrated crop management, and not as a fix-all remedy. It will be dependent on the creation of more cost-effective and stable formulations, as well as explicit economic data provided by large-scale field trials that will demonstrate their economic feasibility to farmers.

## Supplemental Information

10.7717/peerj.21457/supp-1 Supplemental Information 1 Abbreviations of some key words.

## List of abbreviations

6-BA: 6-Benzylaminopurine
ABA: Abscisic Acid
BR(s): Brassinosteroid(s)
bZIP: basic Leucine Zipper
CAT: Catalase
CK(s): Cytokinin(s)
DAS: Days After Sowing
EUI: Elongation of Uppermost Internode
GA/GAs: Gibberellin(s)/Gibberellic Acid
H_2_O_2_: Hydrogen Peroxide
HSP: Heat Shock Protein
IAA: Indole-3-Acetic Acid
IBA: Indole-3-Butyric Acid
JA/MeJA: Jasmonate/Methyl Jasmonate
NAA: Naphthalene Acetic Acid
NUE: Nutrient Use Efficiency
PGR(s): Plant Growth Regulator(s)
RNAi: RNA interference
ROS: Reactive Oxygen Species
SA: Salicylic Acid
SAM: Shoot Apical Meristem
SLAC1: Slow Anion Channel-Associated 1
SOD: Superoxide Dismutase
SnRK2s: SNF1-related protein kinase 2s
WAP: Weeks After Planting

## Additional Information and Declarations

### Data Availability

The following information was supplied regarding data availability:

This is a Literature Review.
