# Supplementary material for: Plant growth regulator applications and mechanisms for boosting rice productivity
Source: PeerJ. 2026 Jul 1;14:e21457. doi: 10.7717/peerj.21457 (PMC13332734; doi:10.7717/peerj.21457)
Supplement: Supplemental Information 1 [file peerj-14-21457-s001.docx]

**List of Abbreviations**

6-BA:   6-Benzylaminopurine

ABA:  Abscisic Acid

BR(s):   Brassinosteroid(s)

bZIP: basic Leucine Zipper

CAT:  Catalase

CK(s):  Cytokinin(s)

DAS:  Days After Sowing

EUI:  Elongation of Uppermost Internode

GA / GAs:  Gibberellin(s) / Gibberellic Acid

H₂O₂:  Hydrogen Peroxide

HSP:  Heat Shock Protein

IAA: Indole-3-Acetic Acid

IBA:  Indole-3-Butyric Acid

JA / MeJA:  Jasmonate / Methyl Jasmonate

NAA:  Naphthalene Acetic Acid

NUE:  Nutrient Use Efficiency

PGR(s):  Plant Growth Regulator(s)

RNAi:  RNA interference

ROS:  Reactive Oxygen Species

SA:  Salicylic Acid

SAM:  Shoot Apical Meristem

SLAC1:  Slow Anion Channel-Associated 1

SOD:  Superoxide Dismutase

SnRK2s:  SNF1-related protein kinase 2s

WAP:  Weeks After Planting
